# The identification of biomarkers for response to omalizumab in adult asthma based on untargeted metabolomic analysis

**DOI:** 10.1097/MD.0000000000047548

**Published:** 2026-02-13

**Authors:** Yajing Li, Yongxiang Zhang, Lingling Zhao, Wei Li, Wei Jia, Hui Ma

**Affiliations:** a Department of Respiratory and Critical Care Medicine, Chest Hospital, Tianjin University, Tianjin, China; b Kindstar Global Precision Medicine Institute, Wuhan, China.

**Keywords:** biomarkers, metabolic profile, metabolomics, omalizumab, severe asthma

## Abstract

There is a paucity of research on biomarkers that can accurately identify patients who are likely to derive benefit from anti-IgE therapy. The present study aimed to identify metabolites associated with the therapeutic response to omalizumab in adult with asthma. We performed global metabolomic profiling of plasma samples obtained from 44 patients with severe asthma prior to initiating omalizumab treatment, employing untargeted metabolomic analysis. Patients were divided into responders or non-responders based on their treatment outcomes. Between-group difference analysis and enrichment analysis were conducted to identify metabolites associated with treatment response. Compared with non-responders, responders exhibited significant up-regulated of 19 metabolites, and down-regulated of 3 metabolites in their plasma. These metabolites may serve as potential indicators for determining which severe asthma patients are likely to benefit from omalizumab. This study highlights the close relationship between the plasma metabolic profile of severe asthma patients and their response to omalizumab. It presents a promising approach for identifying patients who are most likely to benefit from this therapeutic intervention.

## 1. Introduction

Asthma is a chronic respiratory disorder affecting over 300 million individuals globally, characterized by persistent airway inflammation and significant phenotypic heterogeneity. In China alone, epidemiological data indicates that the total number of asthma patients has surpassed 40 million.^[1–3]^ For the majority of cases, inhaled corticosteroids (ICS) in combination with long-acting beta 2-agonist (LABA) effectively improve lung function and reduce disease exacerbations. However, approximately 3% to 10% of patients continue to experience poorly controlled symptoms despite receiving medium-to-high doses of ICS and LABA alongside standardized controller medications for a 6-month period. This subset is classified as having severe asthma according to the Global Initiative for Asthma guidelines.^[4]^ Due to their inadequate response to ICS and LABA, these patients experience diminished quality of life, substantial treatment costs, increased healthcare resource utilization, and elevated rates of mortality and morbidity.^[4,5]^

Anti-IgE therapy utilizes recombinant humanized IgE monoclonal antibodies to target IgE, preventing its binding to effector cells, inhibiting the release of inflammatory mediators, and reducing the levels of high-affinity receptors on the cell surface. This therapeutic approach enables reduction in ICS dosage while simultaneously improving asthma symptoms and lung function.^[6–13]^ Currently, 6 monoclonal antibodies have been approved for asthma treatment: omalizumab, mepolizumab, reslizumab, benralizumab, dupilumab, and tezepelumab. Omalizumab represents the first biologic agent approved by the US Food and Drug Administration specifically for severe asthma. According to the Global Initiative for Asthma guidelines, omalizumab is recommended as a complementary treatment option for patients aged 6 years and older with moderate-to-severe asthma.^[14,15]^ However, under current guidelines, nearly 50% of patients are ineligible for anti-IgE therapy.^[16]^

The identification of sensitive and specific biomarkers to determine patient eligibility for omalizumab treatment could facilitate the rapid formulation of precision treatment strategies, thereby avoiding the increased medical costs and prolonged disease duration associated with ineffective therapies. To this end, we conducted global metabolomic profiling of plasma samples from 44 severe asthma patients prior to omalizumab treatment. Our objective was to analyze and identify metabolites associated with omalizumab response, which would help establish criteria for determining patient suitability for this therapy. This research offers novel insight that may enhance the clinical implementation of targeted treatment and improve therapeutic outcomes.

## 2. Materials and methods

### 2.1. Clinical sample collection

The study subjects involved 44 severe asthmas patients (aged 23–74 years) who were treated at Tianjin Chest Hospital. The inclusion criteria were as follows: patients who demonstrate good compliance and proper inhalation technique but have not achieved asthma control after 3 months or more of regular combined inhalation of ICS and LABA, or those who experience a loss of control following a de-escalation of this treatment^[4]^; patients with no allergy to omalizumab and no history anti-IgE therapy. All patients received maintenance therapy consisting of omalizumab, combined with budesonide and formoterol fumarate powder for inhalation (329 μg bid) and montelukast (10 mg qd) over a period of 16 weeks. Blood samples were collected from the median cubital vein before and after treatment. The samples were centrifuged (1500g, 4 °C, 10 minutes) within 1 hour to isolate plasma, which was then stored at ‐80 °C for subsequent metabolites analysis. This study was approved by the Ethics Committee of the Tianjin Chest Hospital (Approval ID: 2024LW-009), and all participants provided informed consent.

### 2.2. Assessment of treatment efficacy

The 5-item Asthma Control Questionnaire (ACQ-5) is a concise, validated patient-reported outcome questionnaire designed to assess patients’ experiences with asthma over the previous week. It consists of 5 questions that cover the following aspects: nighttime waking by symptoms, symptoms on waking, limitations of daily activities, shortness of breath, and wheezing, on a 7-point scale, where 0 indicates no impairment and 6 signifies maximum impairment. All questions carry equal weight in the overall score. The interpretation of the mean scores is as follows: scores of ≤0.75 indicate well-controlled asthma, scores between >0.75 and ≤1.5 indicate partially controlled asthma, and scores >1.5 indicate not well-controlled asthma. Furthermore, an improvement of ≥0.5 is considered the minimally clinically important difference.^[17,18]^ Based on the change from baseline in the total ACQ-5 score and the mean ACQ-5 score at week 16, the patients were divided into responder group and non-responder group (Table S1, Supplemental Digital Content, https://links.lww.com/MD/R345).

### 2.3. Pulmonary function and fractional exhaled nitric oxide (FeNO)

Patients were instructed to abstain from food, drinks, and alcohol for 1 hour before the test, meanwhile smoking and vigorous exercise were forbidden. FeNO levels were measured in all subjects using the NIOX VERO^®^ (Aerocrine, Solna, Sweden), a hand-held electrochemical device. Participants took a seated position, and held the inspiratory filter. They first exhaled the residual air, then covered the mouth with the filter to inhale in accordance with the test requirements. Flowing this, they exhaled immediately to keep the exhalation flow rate at 50 mL/s (control the exhalation flow rate according to the prompts of the test interface), and started the automatic test and analysis after the exhalation meeting the requirements. Additionally, pulmonary function was assessed using a spirometer, with strict adherence to the operational instructions during the procedure.

### 2.4. Sample preparation and quality control

The thawed samples were mixed on ice and vortexed for 10 seconds. Next, 50 μL of the sample and 300 μL of a 20% acetonitrile methanol solution were added into a microcentrifuge tube, thoroughly mixed, and then centrifugation at 12,000 r/min for 10 minutes. Following centrifugation, 200 μL of the supernatant was collected and stored in ‐20 °C for 30 minutes before being centrifuged at 12,000 r/min for 3min. Finally, 180 μL of the supernatant was transferred for LC–MS analysis. All of these above steps were conducted at 4 °C to ensure the stability of the samples and the accuracy of the analysis. Quality control samples were prepared using the same methods to optimize chromatographic and mass spectrometry conditions, as well as for method validation.

### 2.5. High performance liquid chromatography conditions

The mobile phases used in the analysis consisted of a 0.1% formic acid solution (A) and 0.1% formic acid-acetonitrile solution (B). Gradient elution was carried out using a T3 column (Waters ACQUITY Premier HSS T3 Column, 1.8 µm, 2.1 mm × 100 mm) at a flow rate of 0.4 mL/min. The column temperature was maintained at 40 °C, and the injection volume was set at 4 μL. For a comprehensive overview of the elution conditions, please refer to Table S2, Supplemental Digital Content, https://links.lww.com/MD/R345.

### 2.6. MS conditions

Data acquisition was operated using the information-dependent acquisition mode using Analyst TF 1.7.1 Software (Scitex, Concord, ON, Canada). The values of source parameters are shown in Table S3, Supplemental Digital Content, https://links.lww.com/MD/R345.

### 2.7. Statistical analysis

The original raw file was converted to mzXML format using ProteoWizard software (ProteoWizard, Palo Alto). Peak identification, alignment and retention times analysis were performed using XCMS program. The “SVR” method was utilized to correct peak areas, and peaks with a deletion rate exceeding 50% were excluded from further analysis. Metabolite data was matched with comprehensive public databases, AI databases, and metDNA databases for qualitative mass spectrometry analysis. The processed data underwent multivariate statistical analysis using R software (www.r-project.org), which included principal component analysis and orthogonal partial least squares discriminant analysis (OPLS-DA). This analysis assessed overall metabolic distribution and identified key metabolites based on variable importance in projection (VIP). Student *t* test and fold change (FC) analysis were employed to determine the FC of metabolites between the 2 groups. Metabolites with VIP >1.0, *P* <.05, and FC >2 or FC <0.5 were considered to have significant differences. Identified metabolites were annotated using the Kyoto Encyclopaedia of Genes and Genomes (KEGG) database and mapped to the KEGG pathway database.

## 3. Results

Global metabolomic profiling of plasma samples from 44 patients with severe asthma was performed by untargeted metabolomic analysis. The demographic and clinical characteristics of the participants, together with their blood routine parameters and lung function measurements, are summarized in Table 1 (with detailed information provided in Fig. S1, Supplemental Digital Content, https://links.lww.com/MD/R345). The results indicated that the responder group exhibited significantly higher levels of forced expiratory volume in one second, forced vital capacity, maximal expiratory flow (MEF) at 25%, lymphocytes, eosinophils and IgE compared with the non-responder group (*P* < .05). These findings suggest that these indicators may be associated with the therapeutic response to omalizumab. Conversely, no significant differences were observed between the 2 groups in terms of forced expiratory volume in one second/forced vital capacity ratio, peak expiratory flow, 75% (MEF75) and 50% (MEF50), FeNO, white blood cells, and granulocyte (*P* > .05), indicating limited utility of these parameters in distinguishing treatment response.

**Table 1:** Features of participants.

Parameters	Non-responders	Responders	*P*-value
n	12	32	
Mean age (yr)	46 ± 13	47 ± 13	
Sex (male:female)	3:9	12:20	
Lung function			
FEV1 (L)	1.76 ± 0.62	2.33 ± 0.77	.031
FVC (L)	2.60 ± 0.74	3.28 ± 0.94	.028
FEV1/FVC (%)	67.74 ± 9.54	70.15 ± 10.46	.512
PEF (L/s)	5.15 ± 1.65	6.34 ± 1.96	.075
MEF 75 (L/s)	3.56 ± 1.41	4.25 ± 1.90	.249
MEF 50 (L/s)	1.57 ± 0.89	2.11 ± 1.10	.127
MEF 25 (L/s)	0.43 ± 0.30	0.71 ± 0.41	.024
FeNO (ppb)	53.70 ± 35.31	53.81 ± 42.50	.993
Blood			
WBCs (10^9^/L)	6.22 ± 1.84	7.34 ± 1.91	.102
Granulocyte (10^9^/L)	4.06 ± 1.39	4.17 ± 1.53	.834
Lymphocyte (10^9^/L)	1.52 ± 0.44	2.16 ± 0.65	.001
Eosinophil (10^9^/L)	0.26 ± 0.22	0.56 ± 0.73	.047
IgE (IU/mL)	554.33 ± 434.17	979.41 ± 907.41	.049
ACQ-5 (mean)			
Baseline	3.88 ± 0.75	3.26 ± 0.85	
After treatment	2.93 ± 0.63	1.18 ± 0.43	

ACQ-5 = 5-item Asthma Control Questionnaire, FeNO = fractional exhaled nitric oxide, FEV1 = forced expiratory volume in one second, FVC = forced vital capacity, MEF = maximal expiratory flow, PEF = peak expiratory flow, WBC = white blood cell count.

### 3.1. Data evaluation

The repeatability of metabolite extraction and detection was assessed by comparing the total ion current chromatograms of different quality control samples. The analysis revealed a high degree of overlapping curves in the metabolite-detected total ion current profiles (Fig. S2, Supplemental Digital Content, https://links.lww.com/MD/R345), confirming the stability of both the analytical instrument and the methodology employed.

### 3.2. Multivariate statistical analysis of metabolites

**Figure 1.**
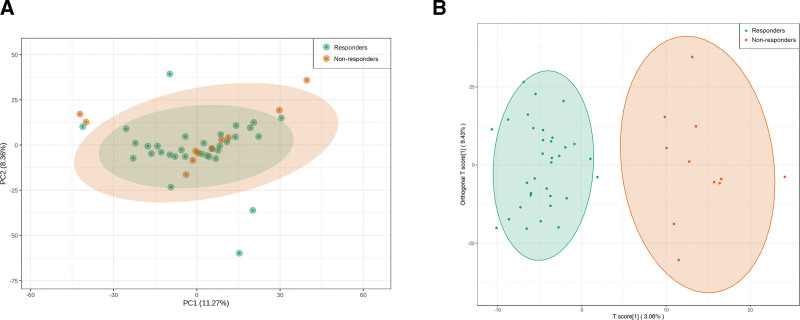
PCA and OPLS-DA on the plasma metabolome of 2 groups of participants. (A) PCA score plot between responders and non-responders. (B) OPLS-DA score plot between responders and non-responders. OPLS-DA = orthogonal partial least squares discriminant analysis; PCA = principal component analysis.

To assess the metabolic profile, principal component analysis was conducted on the plasma metabolome of responders and non-responders. Two principal components were selected, with PC1 and PC2 accounting for 11.27% and 8.36% of the total variance, respectively (Fig. 1A). The plasma metabolomes of 2 groups exhibited distinct clustering and separation. For further discrimination of group-specific metabolomes, supervised OPLS-DA was employed (Fig. 1B). The OPLS-DA model demonstrated clear intragroup sample aggregation and significant intergroup separation, indicating substantial differences in metabolite profiles between responders and non-responders.

### 3.3. Identification of differential metabolites

**Figure 2.**
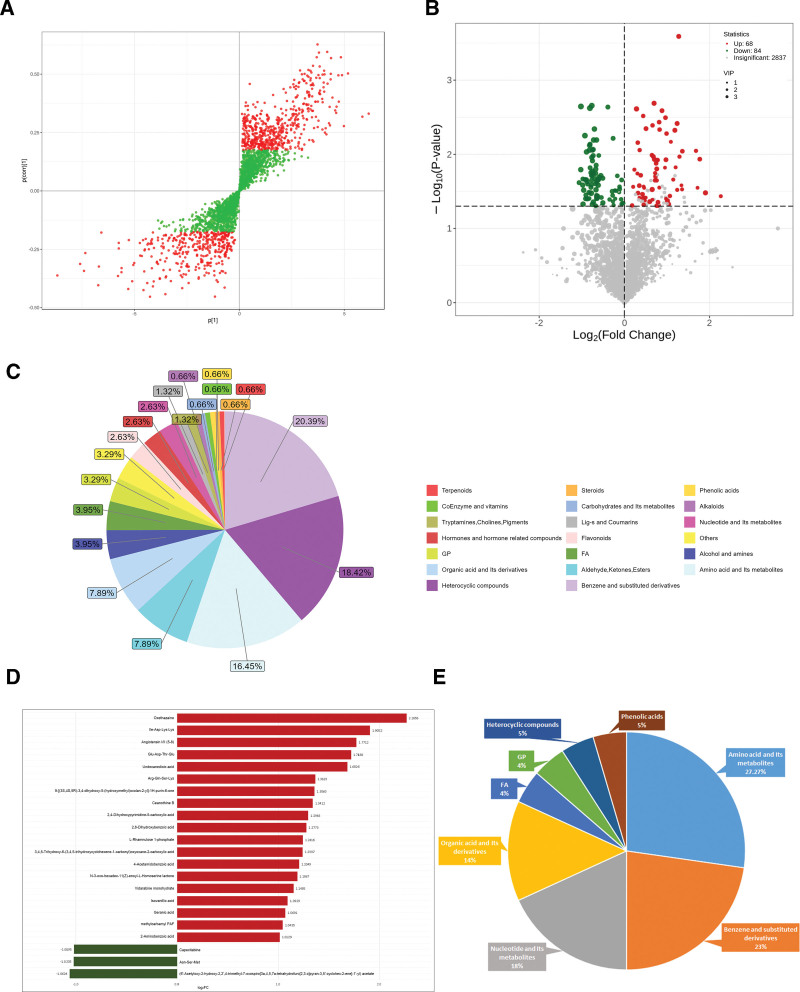
Analysis of differential metabolites between responders and non-responders. (A) OPLS-DA S-plot. The *X*-axis and *Y*-axis represent the covariance and correlation coefficient between the principal component and the metabolite. The farther away from the origin, the more significant the difference between the metabolite is. The red points indicate metabolites with VIP > 1.0, and the green points indicate metabolites with VIP < 1.0. (B) Volcanic map of differential metabolites (VIP > 1.0, *P* < .05). (C) Classification of differential metabolites (VIP > 1.0, *P* < .05). (D) FC bar chart (|log2FC|>1.0). Red indicates up-regulated metabolites, green indicates down-regulated metabolites. (E) Classification of differential metabolites (VIP > 1.0, *P* < .05 and |log2FC|>1.0). FC = fold change, OPLS-DA = orthogonal partial least squares discriminant analysis, VIP = variable importance in projection.

Utilizing the OPLS-DA model, VIP values were calculated to initial screen potential differential metabolites with VIP >1.0 (Fig. 2A). Integrating this with Student *t* test (*P* < .05), a total of 152 differential metabolites were identified between the 2 group, which may serve as potential biomarkers for predicting responsiveness to omalizumab. Among these, 68 metabolites were up-regulated and 84 were down-regulated (Fig. 2B and Fig. S3 Supplemental Digital Content, https://links.lww.com/MD/R345). Notably, benzene and substituted derivatives, heterocyclic compounds, and amino acids and their metabolites constituted a significant proportion of these differential metabolites, accounting for 20.39%, 18.42%, and 16.45% of the total, respectively (Fig. 2C).

Additionally, applying more stringent criteria (FC > 2.0 or <0.5 [|log2FC|>1.0]), 22 differential metabolites were identified, among which only 3 were down-regulated (Fig. 2D). The majority of these differential metabolites were classified as amino acid and their metabolites (27.27%), followed by benzene and substituted derivatives (22.73%) (Fig. 2E).

### 3.4. Metabolic pathway analysis

**Figure 3.**
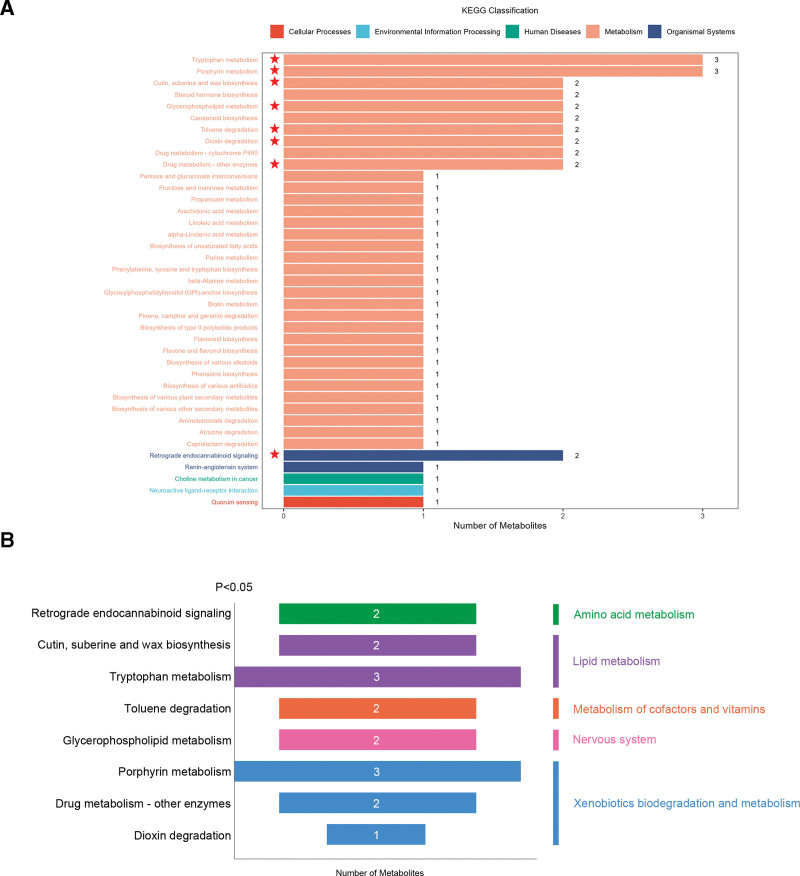
KEGG enrichment analysis of differential metabolites between responders and non-responders. (A) KEGG classification map of differential metabolites. (B) Significant pathways classification map. KEGG = Kyoto Encyclopaedia of Genes and Genomes.

KEGG functional annotation and metabolic pathway enrichment analysis of the differential metabolites revealed their involvement in 39 metabolic pathways (detailed information in Fig. S4, Supplemental Digital Content, https://links.lww.com/MD/R345). According to the KEGG database, the majority of these pathways were categorized under the metabolism category (Fig. 3A). Furthermore, with a significance threshold set at *P* <.05, we identified 8 key metabolic pathways. These pathways included amino acid metabolism, lipid metabolism, the metabolism of cofactors and vitamins nervous system and xenobiotics biodegradation and metabolism (Fig. 3B and Table 2).

**Table 2:** Significant pathways associated with differential metabolites.

Related pathway	*P*-value	Rich factor	Count
Retrograde endocannabinoid signaling	.004	0.1053	2
Cutin, suberine and wax biosynthesis	.008	0.0740	2
Tryptophan metabolism	.008	0.0361	3
Toluene degradation	.023	0.0425	2
Glycerophospholipid metabolism	.032	0.0357	2
Porphyrin metabolism	.038	0.0202	3
Drug metabolism (other enzymes)	.040	0.0317	2
Dioxin degradation	.044	0.0303	1

## 4. Discussion

In China, omalizumab is indicated for the treatment of moderate-to-severe persistent allergic asthma in children (≥6 years old), adolescents and adults whose symptoms remain uncontrolled despite treatment with ICS and LABA. It works by inhibiting the interaction between IgE and its corresponding receptors through specific binding to the 2 Cε3 regions of human IgE. This binding leads to the formation of trimeric or hexametric IgE/anti-IgE complexes, which effectively prevent inflammatory responses and the synthesis of IgE.^[19,20]^ Clinical studies have demonstrated that severe asthma patients receiving omalizumab achieve significant symptoms improvements, including reduced requirements for oral corticosteroids and emergency medications. However, it is important to note that omalizumab is more costly than traditional therapies, and its efficacy is primarily assessed based on changes in patient symptoms and the frequency of acute attacks.^[17,21]^ Currently, there is a lack of effective indicators to accurately identify which patients are most suitable for omalizumab treatment and to guide the formulation and adjustment of clinical regimens.

In recent years, various biomarkers for asthma have been increasingly integrated into clinical practice. For example, elevated sputum eosinophil levels are closely associated with the responsiveness to inhaled ICS, and monitoring eosinophil levels can provide critical insights into a patient’s response to corticosteroid therapy.^[22,23]^ Additionally, FeNO levels reflect eosinophilic inflammation and serve as a measure of asthma efficacy, with high clinical value for asthma diagnosis and prediction of corticosteroids responsiveness.^[24,25]^ The periostin protein, recognized as a biomarker for Th2-type asthma, has been shown to predict patient responses to various treatments, including inhaled ICS, Th2-targeted monoclonal antibodies, anti-IL-13 monoclonal antibodies, and anti-IgE monoclonal antibodies.^[26–28]^ In our study, lymphocytes, eosinophils and IgE levels were significantly higher in the responder group compared to the non-responder group (*P* < .05), suggesting that these indicators may be associated with omalizumab efficacy. Conversely, FeNO, white blood cells, and granulocytes showed no significant differences between the 2 groups (*P* > .05). With the advancement of high-throughput technologies, particularly metabolomics, the potential to identify clinical biomarkers linked to asthma therapeutic effects has expanded. Alterations in metabolite profiles may correlate closely with asthma onset, phenotype, severity, and treatment response.^[29–34]^ In light of this, we conducted an untargeted metabolomics analysis of plasma samples from severe asthma patients to identify metabolites associated with omalizumab response.

In this study, we analyzed plasma samples from 44 patients with severe asthma and identified 152 metabolites (VIP > 1.0, *P* < .05) associated with their response to omalizumab treatment. The identification of these metabolites prompted us to explore their potential mechanistic roles. We hypothesized that these differential metabolites might influence the patients’ response to omalizumab by modulating the IgE pathway, affecting mast cell activity, and regulating the Th2-type inflammatory response. Firstly, amino acids and their metabolites play a significant role in asthma regulation. Research has indicated that certain amino acids, such as arginine and glutamic acid, serve as precursors for nitric oxide synthesis and may also regulate mast cell activity by altering the local immune environment.^[35–39]^ Changes in the levels of these amino acids could directly impact IgE synthesis and its binding to mast cells, thereby reflecting varying degrees of patient response to omalizumab. Existing literature suggests^[40–42]^ that alterations in amino acid metabolism are closely linked to the pathophysiology of asthma, particularly in the context of Th2-type inflammation, which supports our hypothesis. Secondly, lipid metabolism is also crucial in the validation response of asthma. Specific lipids, such as arachidonic acid and its derivatives, are converted into bioactive substances during the inflammatory process, capable of activating mast cells and promoting IgE-mediated allergic reactions.^[43]^ Our findings revealed differences in lipid metabolism between patients who responded to omalizumab and those who did not, indicating that these metabolites may play a significant role in regulating asthma inflammation and treatment responses. Therefore, future research should focus on exploring the specific mechanisms by which these metabolites regulate immune and inflammatory responses in asthma.

Despite the valuable findings from our research, several limitations must be acknowledged. Firstly, the small sample size, particularly in the non-responder group, may introduce instability in the statistical results, which could restrict the generalizability of our findings. Secondly, many patients were concurrently receiving standard treatments, such as inhaled ICS, which may have influenced metabolic changes and the patients’ treatment responses. Additionally, as a single-center study, the uniqueness of our sample selection may limit the applicability of our results. Future research should involve larger-scale, multicenter studies to validate our findings. It is important to note that our conclusions are preliminary, aimed at generating hypotheses and establishing a foundation for subsequent investigations. Our study indicates that amino acid and lipid metabolism may be associated with the treatment response of asthma patients to omalizumab; however, these results require confirmation through larger, multicenter, prospective cohorts. Moving forward, research should prioritize the development of targeted detection methods to more effectively assess the applicability of omalizumab treatment in clinical practice. This approach will help optimize individualized treatment strategies for asthma patients.

## Author contributions

**Conceptualization:** Yajing Li.

**Data curation:** Lingling Zhao, Wei Li.

**Formal analysis:** Wei Jia.

**Funding acquisition:** Hui Ma.

**Methodology:** Yongxiang Zhang.

**Validation:** Lingling Zhao, Wei Li.

**Writing – original draft:** Yajing Li, Yongxiang Zhang.

**Writing – review & editing:** Hui Ma.

## Supplementary Material


