# Supplementary material for: The identification of biomarkers for response to omalizumab in adult asthma based on untargeted metabolomic analysis
Source: Medicine (Baltimore). 2026 Feb 13;105(7):e47548. doi: 10.1097/MD.0000000000047548 (PMC12908810; doi:10.1097/MD.0000000000047548)

***Supplementary Material***

### 1. Table

**Table S1 Statistical table of patients' ACQ-5 scores.**

| Non-responders | | | Responders | | |
| --- | --- | --- | --- | --- | --- |
| Baseline | After treatment | ACQ-5 change | Baseline | After treatment | ACQ-5 change |
| 2.3 | 2.4 | 0.1 | 3.4 | 1.3 | -2.1 |
| 3.5 | 3.1 | -0.4 | 3 | 1 | -2.0 |
| 3.3 | 2.9 | -0.4 | 2.6 | 1.1 | -1.5 |
| 4 | 3.6 | -0.4 | 4 | 1 | -3.0 |
| 2.2 | 2.5 | 0.3 | 1.5 | 0.8 | -0.7 |
| 3 | 2.9 | -0.1 | 2.5 | 0.94 | -1.56 |
| 2.5 | 3 | 0.5 | 3.3 | 1.4 | -1.9 |
| 2.7 | 2.4 | -0.3 | 4 | 1 | -3.0 |
| 2 | 1.8 | -0.2 | 3.5 | 1.6 | -1.9 |
| 4 | 4.1 | 0.1 | 2.5 | 1.2 | -1.3 |
| 4.2 | 3.8 | -0.4 | 3 | 1.25 | -1.75 |
| 2.3 | 2.6 | 0.3 | 2.1 | 0.87 | -1.23 |
|  |  |  | 1.8 | 0.9 | -0.9 |
|  |  |  | 5 | 0.95 | -4.05 |
|  |  |  | 4 | 0.88 | -3.12 |
|  |  |  | 2.5 | 1.1 | -1.4 |
|  |  |  | 3 | 1.3 | -1.7 |
|  |  |  | 2.8 | 0.6 | -2.2 |
|  |  |  | 3.6 | 0.8 | -2.8 |
|  |  |  | 2.3 | 1.1 | -1.2 |
|  |  |  | 3.3 | 1.3 | -2.0 |
|  |  |  | 3.5 | 0.9 | -2.6 |
|  |  |  | 2.9 | 0.6 | -2.3 |
|  |  |  | 4 | 1.7 | -2.3 |
|  |  |  | 3.5 | 1.4 | -2.1 |
|  |  |  | 4.2 | 2.1 | -2.1 |
|  |  |  | 5.3 | 2.3 | -3.0 |
|  |  |  | 2.4 | 0.5 | -1.9 |
|  |  |  | 3.1 | 1.2 | -1.9 |
|  |  |  | 3.7 | 1.1 | -2.6 |
|  |  |  | 3.6 | 1.2 | -2.4 |
|  |  |  | 4.4 | 2.3 | -2.1 |

**Table S2 Elution conditions of HPLC.**

| **Time (min)** | **A (%)** | **B (%)** |
| --- | --- | --- |
| 0 | 95 | 5 |
| 2.0 | 80 | 20 |
| 5.0 | 40 | 60 |
| 6.0 | 1 | 99 |
| 7.5 | 1 | 99 |
| 7.6 | 95 | 5 |
| 10 | 95 | 5 |

**Table S3 The values of the source parameters of MS.**

| **Parameters** | **ESI^+^** | **ESI^-^** |
| --- | --- | --- |
| Duration | 10 (min)  50 (psi)  60 (psi)  35 (psi)  15 (V)  50~1000 (Da)  25~1000 (Da)  0.2 (s)  0.04 (s)  18 | |
| Ion Source Gas1 |  |  |
| Ion Source Gas2 |  |  |
| Curtain Gas |  |  |
| Collision Energy Spread |  |  |
| MS1 TOF Masses |  |  |
| MS2 TOF Masses |  |  |
| MS1 Accumulation time |  |  |
| MS2 Accumulation time |  |  |
| Candidate ions |  |  |
| Delustering Potential | 60 (V) | -60 (V) |
| MS1 Collision Energy | 10 (V) | -10 (V) |
| MS2 Collision Energy | 30 (V) | -30 (V) |
| Ion Spray Voltage | 5000 (V) | -4000 (V) |
| Temperature | 550 (°C) | 450 (°C) |
| Exclude former target ions | Always, For 3 seconds, After 3 occurrences | |

### 2. Figure

**Figure S1.** Details of participant characteristics, blood routine tests, and lung function were obtained.


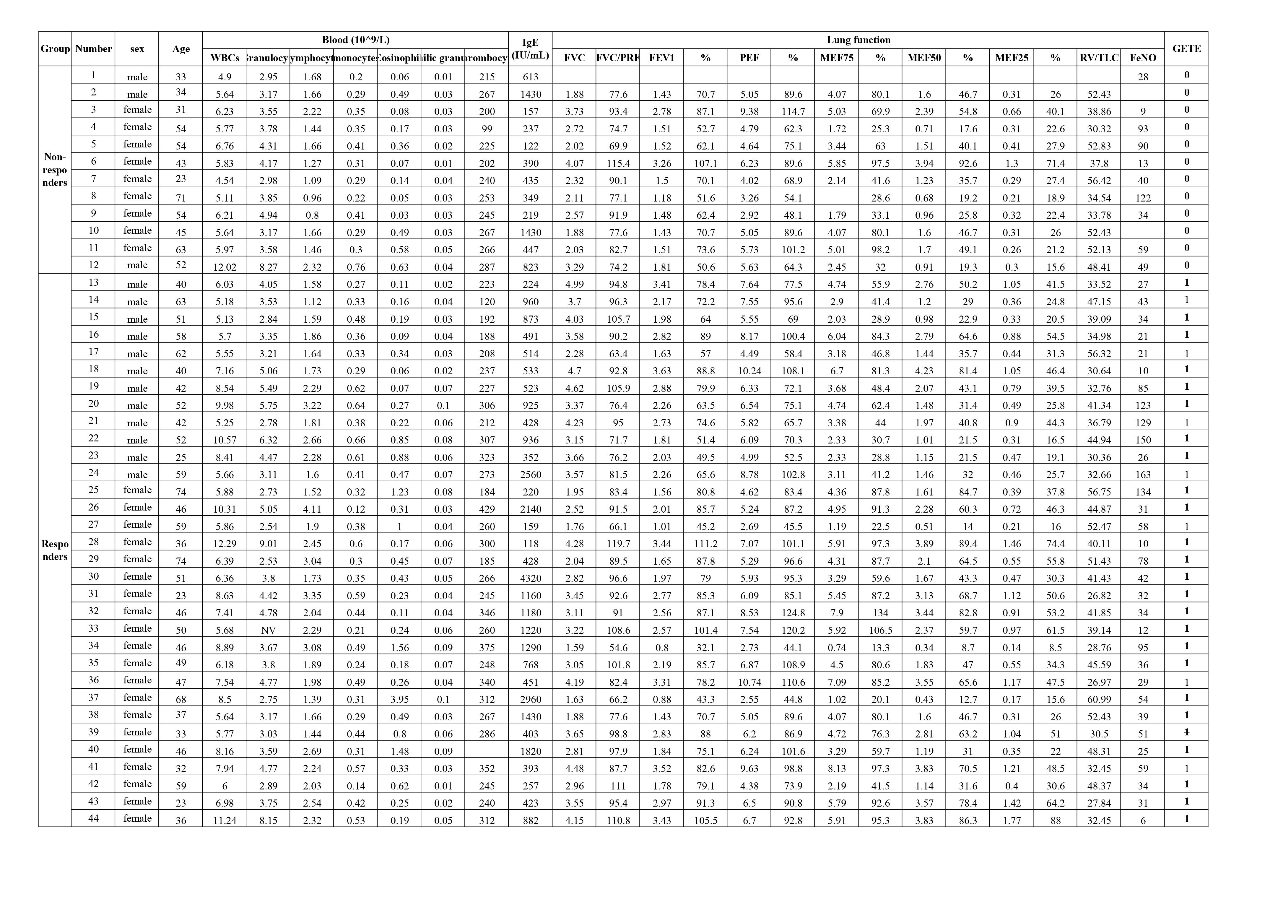


**Figure S2.** Total ion current chromatograms of metabolites in both groups.


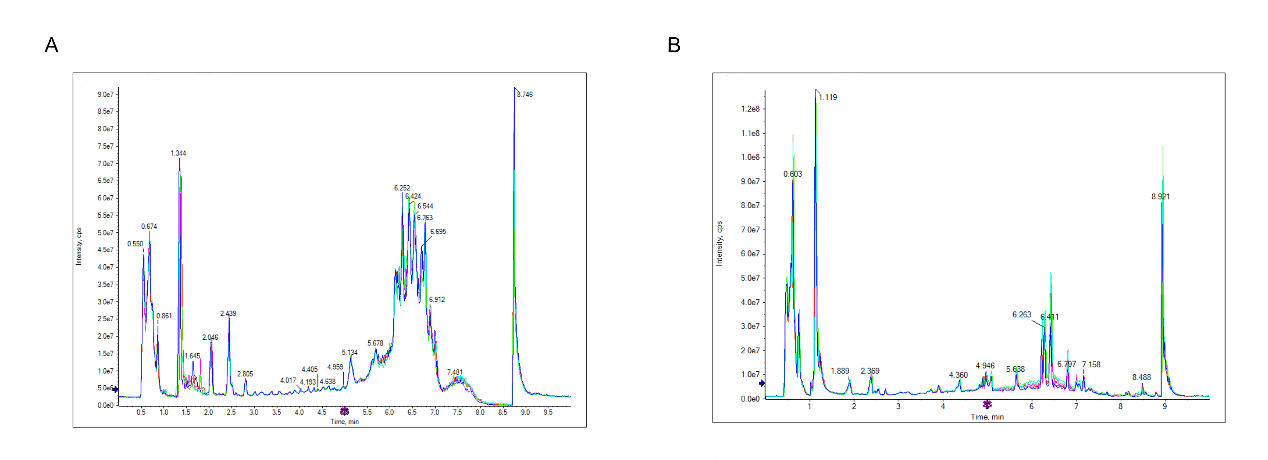


**Figure S3.** Differential metabolite analysis of the two groups of patients.


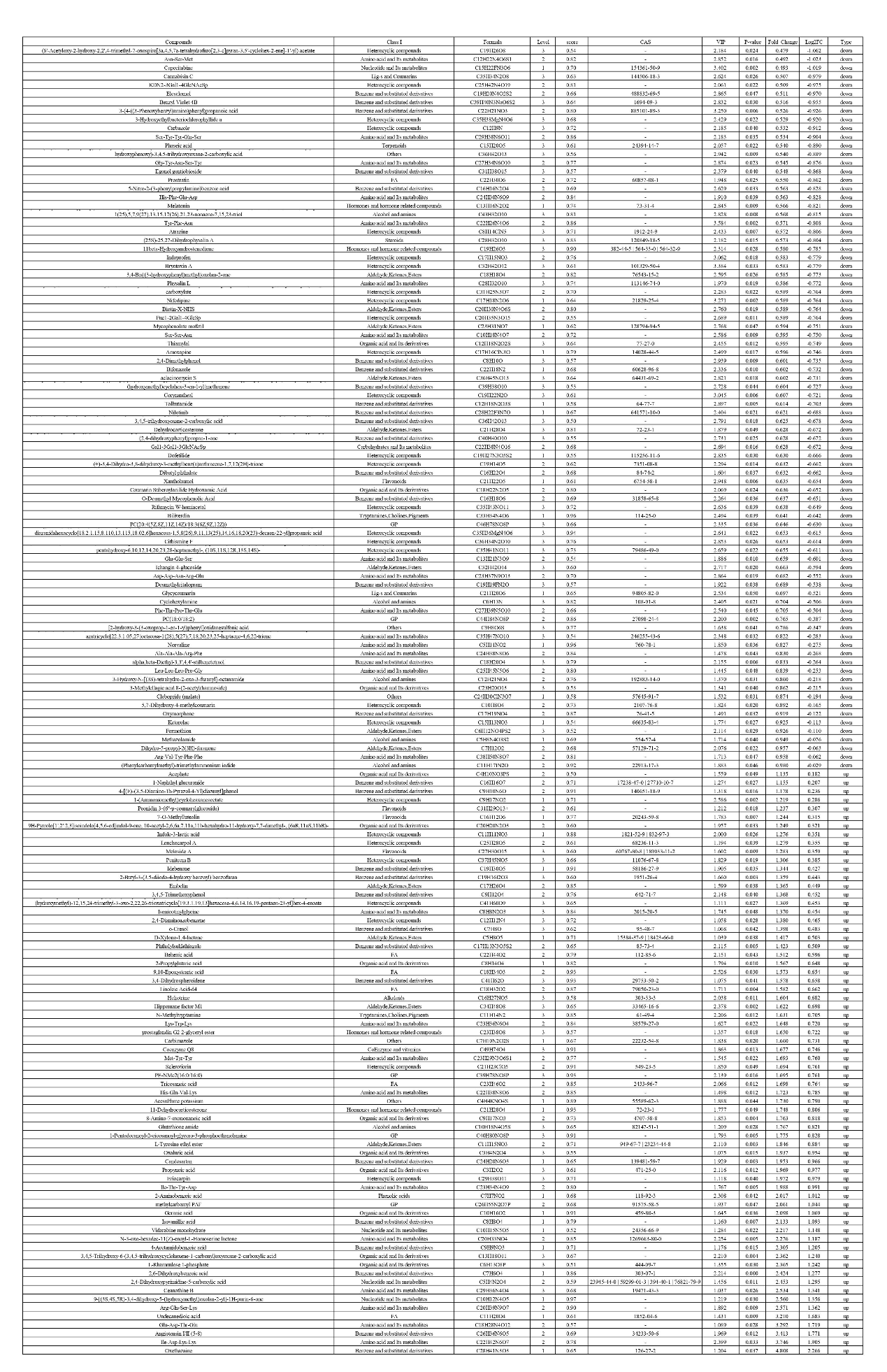


**Figure S4.** Results of KEGG functional annotation and metabolic pathway enrichment analysis of differential metabolites.


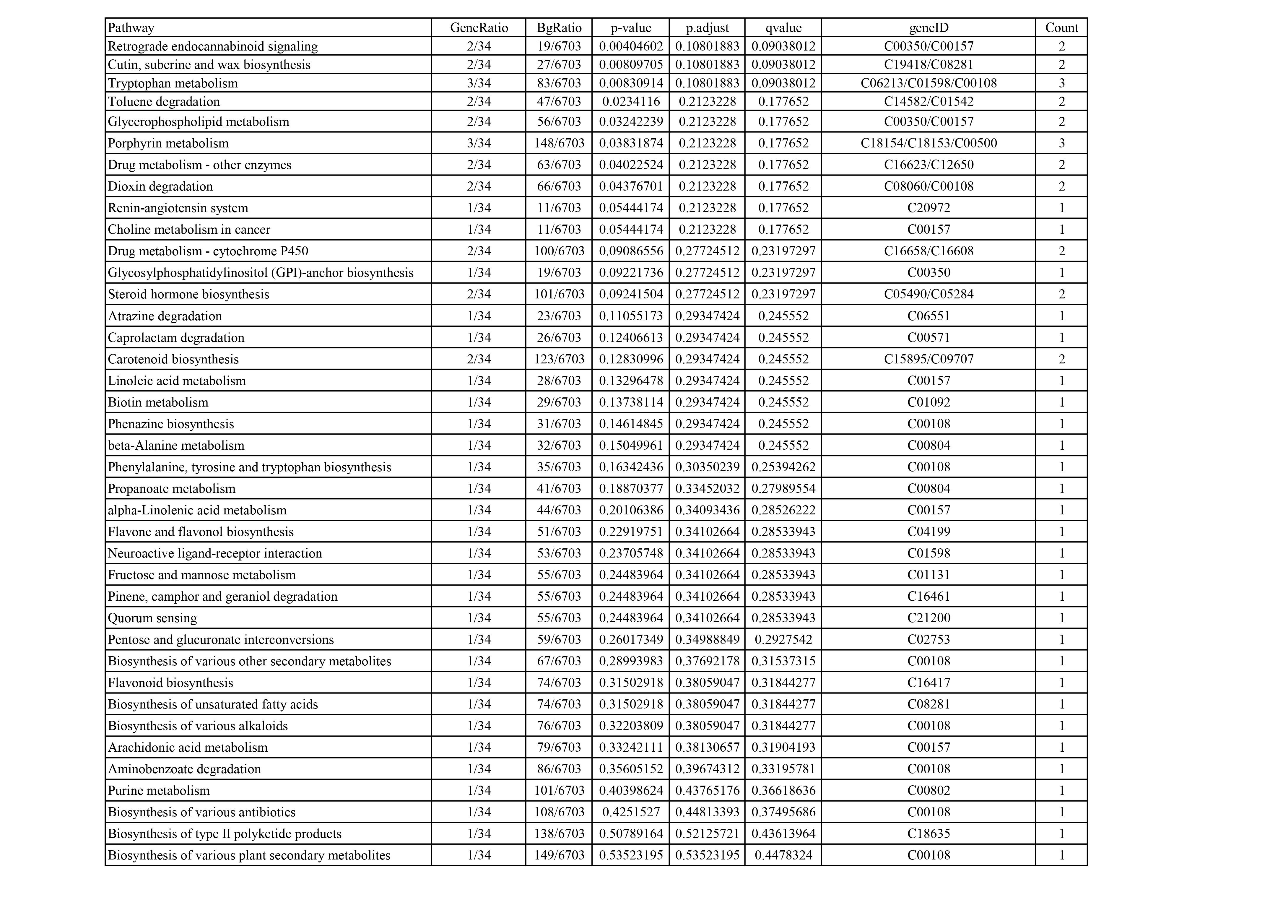

Supplement: Supplementary file 1 [file medi-105-e47548-s001.docx]
